# The epidemiology of overtransfusion of red cells in trauma resuscitation patients in the context of a mature massive transfusion protocol

**DOI:** 10.1007/s00068-021-01678-0

**Published:** 2021-04-30

**Authors:** Timothy Cowan, Natasha Weaver, Alexander Whitfield, Liam Bell, Amanda Sebastian, Stephen Hurley, Kate L. King, Angela Fischer, Zsolt J. Balogh

**Affiliations:** 1 Department of Emergency Medicine, John Hunter Hospital, Newcastle, NSW Australia; 2 Hunter Medical Research Institute, Newcastle, NSW Australia; 3 Department of Traumatology, John Hunter Hospital, Newcastle, NSW Australia; 4 The University of Newcastle, Newcastle, NSW 2310 Australia

**Keywords:** Transfusion, Shock, Trauma, Massive transfusion protocol, Overtransfusion, Resuscitation

## Abstract

**Purpose:**

Packed red blood cell (PRBC) transfusion remains an integral part of trauma resuscitation and an independent predictor of unfavourable outcomes. It is often administered urgently based on clinical judgement. These facts put trauma patients at high risk of potentially dangerous overtransfusion. We hypothesised that trauma patients are frequently overtransfused and overtransfusion is associated with worse outcomes.

**Methods:**

Trauma patients who received PRBCs within 24 h of admission were identified from the trauma registry during the period January 1 2011–December 31 2018. Overtransfusion was defined as haemoglobin concentration of greater than or equal to 110 g/L at 24 h post ED arrival (± 12 h). Demographics, injury severity, injury pattern, shock severity, blood gas values and outcomes were compared between overtransfused and non-overtransfused patients.

**Results:**

From the 211 patients (mean age 45 years, 71% male, ISS 27, mortality 12%) who met inclusion criteria 27% (56/211) were overtransfused. Patients with a higher pre-hospital systolic blood pressure (112 vs 99 mmHg *p* < 0.01) and a higher initial haemoglobin concentration (132 vs 124 *p* = 0.02) were more likely to be overtransfused. Overtransfused patients received smaller volumes of packed red blood cells (5 vs 7 units *p* = 0.049), fresh frozen plasma (4 vs 6 units *p* < 0.01) and cryoprecipitate (6 vs 9 units *p* = 0.01) than non-overtransfused patients.

**Conclusion:**

More than a quarter of patients in our cohort were potentially given more blood products than required without obvious clinical consequences. There were no clinically relevant associations with overtransfusion.

## Background

Approximately 5% of trauma patients require transfusion of blood products [1]. The decision to transfuse packed red blood cells in shocked trauma patients is a complex one that is not readily amenable to rigid guidelines or decision rules. Emergency and trauma clinicians are faced with the potentially divergent challenges of not causing harm by unnecessary intervention, providing adequate and appropriate resuscitation, and overseeing the stewardship of a precious and expensive resource. The situation is further complicated by the fact there is no consensus definition of what constitutes overtransfusion. Reported rates of overtransfusion in trauma vary from 6.5 to 62% [2–4], but with such diverse definitions the true rate remains uncertain. This study was undertaken to determine overtransfusion rates in a mature trauma system with functional massive transfusion protocol (MTP) for 15 years and determine any associated factors that might predict patients at increased risk. We hypothesised that overtransfusion is frequent and especially among patients who receive prehospital blood and as a result of MTP.

## Methods

This retrospective study was performed at John Hunter Hospital (Royal Australasian College of Surgeons verified level 1 trauma centre in Newcastle, Australia) based on the institutional prospectively maintained trauma registry. All patients who received packed red blood cell transfusion within the first 24 h of their trauma admission were identified between January 1, 2011 and December 31, 2018. Only primary trauma centre admissions were included, transferred patients were excluded. The hospital medical records were reviewed to determine the haemoglobin concentration at 24 h post admission. Data collectors were blinded to the study question and entered data on a standardised form [5].

Overtransfusion was defined as a subsequent haemoglobin greater than or equal to 110 g/L at 24 h (± 12 h). This definition was intentionally conservative and allowed a target haemoglobin of 100g/L with a 10% buffer to account for the known challenges in predicting transfusion requirements in early trauma resuscitation. A similar cut-off has been used in other studies [6].

Data collected included demographics, pre-hospital and ED vital signs, initial blood gas results, initial and subsequent haemoglobin levels, transfused blood products including whether massive transfusion protocol was activated, injury severity score, multiple organ failure and survival to discharge. Continuous variables are presented as means and standard deviations or medians with interquartile range. Categorical variables are presented as frequencies and percentages. *p* values < 0.05 were considered significant. Characteristics of overtransfused patients were compared to non-overtransfused patients using logistic regression for categorical or normal variables or the Wilcoxon-Mann–Whitney test for nonparametric variables. SAS statistical software (SAS Institute, Cary NC, USA) was used for analysis.

## Results

**Fig. 1:**
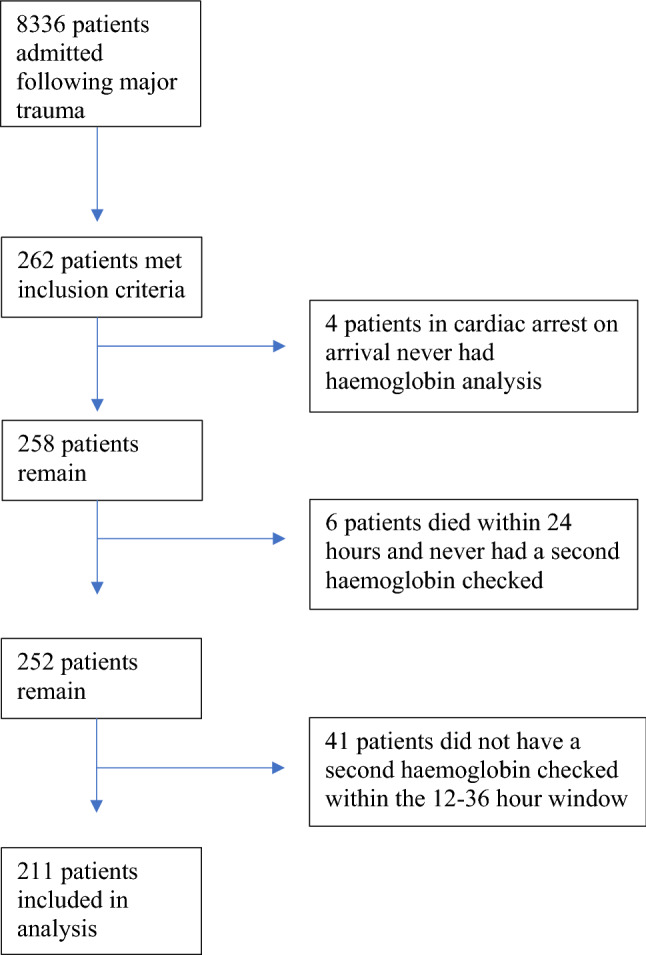
Flowchart of included patients

Two hundred and sixty two patients were identified that met the inclusion criteria within the study period. Of these, four patients who were in cardiac arrest on arrival to ED did not have a haemoglobin analysis. A further six patients died within the first 24 h and did not have a second haemoglobin analysis. Of the remaining 252 patients, 211 had a haemoglobin analysis in the 12–36 h window and served as the study population (Fig. 1).

56 of these 211 (27%) met the study definition of overtransfusion, Table 1, outlines the demographic characteristics of the two groups.

**Table 1:** Demographic and clinical features

Variable	Class	Hb > = 110 at 24 h	Hb < 110 at 24 h	Odds ratio (95% CI)	*p* value
*N*		56	155		
Age in years		44.6 (21.8)	44.8 (21.6)	1.00 (0.98–1.01)	0.95
Injury Severity Score (ISS)		29.3 (13.0)	28.4 (13.9)	1.00 (0.98–1.03)	0.69
Length of stay (LOS)		24.1 (25.0)	36.4 (53.5)	0.99 (0.98–1.00)	0.10
Time in ED (hours)		2.9 (2.4)	3.4 (2.8)	0.93 (0.82–1.05)	0.21
Discharge status	Died	9 (16.0%)	16 (10%)		
	Survived	47 (84%)	139 (90%)	0.60 (0.25–1.45)	0.26
Gender	Female	15 (27%)	47 (30%)		
	Male	41 (73%)	108 (70%)	1.19 (0.60–2.36)	0.62
Serious head injury	No	48 (86%)	123 (79%)		
	Yes	8 (14%)	32 (21%)	0.64 (0.28–1.49)	0.30
Massive transfusion protocol (MTP)	No	24 (43%)	54 (35%)		
	Yes	32 (57%)	101 (65%)	0.71 (0.38–1.33)	0.29
Pre-hospital provider	Ambulance service NSW	47 (84%)	131 (85%)		
	Retrieval service	9 (16%)	24 (15%)	1.05 (0.45–2.41)	0.92
Primary injury type	Blunt	45 (80%)	136 (88%)		
	Penetrating	11 (20%)	19 (12%)	1.75 (0.77–3.95)	0.18
Post-ED disposition	Intensive care unit	27 (48%)	54 (35%)		
	Operating suite	28 (50%)	83 (54%)	0.67 (0.36–1.27)	0.22
	Ward	1 (1.8%)	18 (12%)	0.11 (0.01–0.88)	0.037

Of the 211, the mean age was 44.7 years and 149/211 (71%) were male. 25/211 patients (12%) did not survive to hospital discharge. The median ISS across this cohort was 27 (IQR 17–38) and 30/211 (14%) of patients experienced a penetrating mechanism of injury. 40/211 (19%) had a severe head injury. When examining AIS by region, overtransfused patients were significantly more likely to have an abdominal AIS > 2 and significantly less likely to have an extremity AIS > 2. Otherwise injury patterns were similar (Table 2). 111/211 (53%) patients were taken directly from ED to the operating room. Median base deficit was 4.4 (IQR 1.7–8.4) and the median number of units of packed cells administered was 4.5 (IQR 3–8).

**Table 2:** Abbreviated injury score (AIS) by region

Body region	Hb > = 110 at 24 h (*n* = 56)	Hb < 110 at 24 h (*n* = 155)	OR (95% CI)	*p* value
Head/neck Score > 2	14 (25%)	47 (30%)	0.77 (0.39–1.53)	0.45
Face Score > 2	4 (7%)	3 (2%)	3.90 (0.94–16.09)	0.08**
Chest Score > 2	33 (59%)	88 (57%)	1.09 (0.59–2.02)	0.78
Abdomen Score > 2	29 (52%)	45 (29%)	2.63 (1.41–4.90)	0.002
Extremities Score > 2	19 (34%)	88 (57%)	0.39 (0.21–0.74)	0.003
External Score > 2	2 (4%)	10 (6%)	0.54 (0.00–2.27)	0.34**

The confidence intervals provided are based on cornfield approximations (which tend to perform worse when there are small cell counts)

**For small sample sizes, where expected cell counts are smaller than 5 (such as Face Score and External Score) Fisher's exact test is used instead of the Chi-squared test

133/211 (63%) patients had a massive transfusion protocol (MTP) activated. Individual components transfused are outlined in Table 3.

**Table 3:** Blood products administered

Variable	Hb > = 110	Hb < 110	*p* value
Packed red blood cells (units)	4 (6)	5 (6)	0.08
Fresh frozen plasma (units)	2 (3.5)	4 (5)	0.010
Cryoprecipitate (units)	4.5 (9)	6 (10)	0.028
Platelets (pools)	0.0 (0)	0 (1)	0.18

Table 3 demonstrates that while overtransfused patients received significantly less fresh frozen plasma and cryoprecipitate than the normally transfused group, red cell transfusion volumes were not significantly different. The significant difference in haemoglobin levels on initial blood gas between groups persisted at 24 h—the median haemoglobin in the overtransfused group was 121 g/L (115.5–131) compared with 93 g/L (86–100) (*p* < 0.05).

None of the differences in massive transfusion protocol activation (24% overtransfused vs 31% not overtransfused), length of stay (24 vs 36 days), rates of serious head injury (20% vs 28%), penetrating injury mechanism (37% vs 25%) or in-hospital mortality (36% vs 25%) reached statistical significance.

Of the 25 patients who died, 18 had a severe brain injury. Two patients died suddenly on the ward post ICU discharge, and the remaining five died from multiple organ failure or were palliated due to age and comorbidities. No patients died due to exsanguination in hospital.

The balance of blood products transfused was not significantly associated with overtransfusion. The ratio of packed red blood cells to fresh frozen plasma was 1.74 (SD 0.92) in overtransfused compared to 1.48 (SD 0.87) in not (OR 1.35 (95% CI 0.94–1.96), *p* = 0.1079. The ratio of packed red blood cells to cryoprecipitate was 1.18 (0.81) in overtransfused compared to 0.9 (0.61) in not (OR 1.72 (0.97–3.05 *p* = 0.07). The ratio of packed red blood cells to pools of platelets was 7.73 (3.48) in overtransfused compared to 8.87 (4.06) in not (OR 0.92 (0.78–1.09), *p* = 0.35.

Of note there was no association between overtransfusion and being brought to hospital by a medical retrieval service (critical care doctor and paramedic, travelling by road or helicopter) who carry packed red blood cells. Only 31 patients (16%) were brought to hospital by retrieval service. Of these 31, 19 received blood pre-hospital.

Tables 4 and 5 describe the observations recorded pre-hospital and on arrival in the ED.

**Table 4:** Pre-hospital vital signs

Variable	Hb > = 110	Hb < 110	Odds ratio (95% CI)	*p* value
Systolic blood pressure (SBP) (mmHg)	111.8 (29.82)	99.02 (27.39)	1.02 (1.00–1.03)	0.008
Diastolic blood pressure (DBP) (mmHg)	67.65 (20.13)	62.89 (18.05)	1.01 (0.99–1.04)	0.22
Median glasgow coma scale (IQR)	13 (6)	14 (6)		0.19
Heart rate (bpm)	105.39 (28.98)	105.54 (30.66)	1.00 (0.99–1.01)	0.98
Respiratory rate (per minute)	24.64 (9.90)	24.36 (9.07)	1.00 (0.97–1.04)	0.86
Oxygen saturation (SaO2)	90.43 (15.31)	92.12 (11.51)	0.99 (0.97–1.01)	0.44

**Table 5:** Initial vital signs in ED

Variable	Hb > = 110	Hb < 110	Odds ratio (95% CI)	*p* value
Systolic blood pressure (SBP) (mmHg)	108.73 (31.16)	106.25 (29.78)	1.00 (0.99–1.01)	0.60
Diastolic blood pressure (DBP) (mmHg)	65.35 (22.68)	67.83 (21.97)	0.99 (0.98–1.01)	0.48
Glasgow coma scale (GCS)	14 (12)	14 (8)		0.23
Heart rate (bpm)	111.79 (28.48)	106.24 (29.93)	1.01 (1.00–1.02)	0.23
Respiratory rate (per minute)	21.61 (7.02)	21.98 (7.94)	0.99 (0.95–1.04)	0.76
Oxygen saturation (SaO2)	95.76 (6.24)	96.52 (5.30)	0.98 (0.93–1.03)	0.39
Temperature (degrees Celsius)	35.67 (1.38)	35.61 (1.06)	1.04 (0.79–1.38)	0.78

A higher systolic blood pressure pre-hospitally was associated with overtransfusion. The prehospital mean systolic BP in the overtransfused patients was 111 mmHg compared to 99 mmHg in those not overtransfused (*p* = 0.008). This difference was not found on the initial vital signs taken in the ED, where systolic BP was 109 mmHg compared with 106 mmHg (*p* = 0.60).

Table 6 shows the initial blood gas findings. Blood gas values on arrival to ED were similar for the two groups, apart from haemoglobin concentration which was significantly higher in the overtransfusion group by 7.87 g/l with 95% CI 1.25–14.50 *p* = 0.020.

**Table 6:** Blood gas values on arrival to ED

Variable	Hb > = 110	Hb < 110	Odds ratio (95% CI)	*p* value
Base deficit/base excess (mmol/L)	−5.54 (5.52)	−5.29 (5.94)	0.99 (0.94–1.05)	0.79
Haemoglobin concentration (g/L)	132.15 (24.52)	124.27 (20.06)	1.02 (1.00–1.03)	0.02
Bicarbonate (HCO3-) (mmol/L	22.44 (4.14)	22.04 (4.29)	1.02 (0.95–1.10)	0.55
Lactate concentration (mmol/L)	4.85 (2.95)	4.78 (3.31)	1.01 (0.91–1.11)	0.89
Partial pressure carbon dioxide (PaCO2) (mmHg)	54.59 (13.19)	54.31 (23.33)	1.00 (0.99–1.02)	0.93
Partial pressure oxygen (PaO2) (mmHg)	56.13 (60.75)	56.2 (62.03)	1.00 (0.99–1.01)	0.99

A subgroup (66/211) of patients were high risk for multiple organ failure (MOF) and met inclusion criteria (ISS > 15, AIS head < 3, age > = 16, admission of 48hrs + to ICU) for our institutional prospective MOF database. Of the patients who developed MOF, there was no significant difference in rates of overtransfusion. 21/66 (32%) were overtransfused compared to 45/66 (68%) (*p* = 0.26).

When comparing available biochemical markers of perfusion at 24 h post ED arrival (pH, base excess and lactate) there were no clinically significant differences.

## Discussion

Comparing overtransfusion rates across trauma centres is difficult when the definitions vary from study to study. Our chosen definition of overtransfusion was a haemoglobin concentration of 110 g/L at 24 h following ED arrival. Our local trauma surgeons target a haemoglobin of 100 g/L during trauma resuscitation, which aligns with the recommended endpoint for red cell transfusion in the American College of Surgeons Massive Transfusion in Trauma guidelines. [7] The rationale behind an additional 10 g/L allowance is to recognise that the decision to transfuse is time critical and is often made without the benefit of following trends in haemodynamic stability or perfusion. Using this definition, the overtransfusion rate in our study was 27%.

Over the study period there has been an increasing trend towards restrictive transfusion in critical care settings. Analysis of 200 patients in the trauma subgroup of the TRICC trial did not demonstrate a mortality benefit when using an Hb target of 100 g/L as opposed to 70 g/L [8]. The recently updated European guidelines on bleeding in trauma recommend a target Hb of 70–90 g/L [9]. A logical conclusion might be that we need to more carefully analyse our transfusion practices to further determine the risk factors for preventable transfusion. However, it is important to also consider, where the equipoise lies between overtransfusion and undertransfusion. Undertransfusion in haemorrhagic shock and coagulopathy may well carry greater risk to the patient, so until transfusion triggers and monitoring become more accurate it may be reasonable to accept a slight rate of overtransfusion.

There is also a financial cost to consider: a unit of packed red blood cells costs $428.05 [10]; however, the cost of administration of that unit can mean the health service pays 3–4 times that amount per unit transfused.

As primary retrieval services (medically escorted transfer from site of injury to trauma centre) mature in Australia, increasing pre-hospital blood product administration is likely to occur, especially with novel blood products that can be freeze dried to facilitate transport and storage. This shift to earlier haemostatic resuscitation may influence in-hospital transfusion requirements, especially in health services spread over large geographic areas.

In a population of non-trauma patients Barr et al. have reported a greater risk of overtransfusion in women due to their reduced body volume [4]. In trauma patients, overtransfusion has also been significantly associated with lower injury severity and greater haemodynamic stability [6]. Haemodynamic assessment has numerous elements, but we did identify that patients with a higher pre-hospital systolic BP were more likely to be overtransfused. Sisak et al. found that overtransfusion was more likely in patients receiving low volume transfusion [11].

Despite physiologic similarities at ED presentation, the overtransfused group may represent patients who achieved early haemorrhage control and thus needed less blood products. Their injury severity may have been contributed to by non-haemorrhagic injuries.

Intra-abdominal bleeding may be more likely to trigger transfusion in ED given the more complex decision making needed around source control, and the overtransfused group had proportionately more abdominal injury.

While the overtransfused group had a higher haemoglobin level on ED arrival, it is well established that this is not a reliable marker to predict transfusion requirements. Moreover, the difference of 132 g/L vs 124 g/L is not clinically significant.

Numerous scoring systems have tried to predict MTP requirement. A systematic review of various of these tools concluded that while performance was reasonable, their dependence on local resourcing meant it was not possible to recommend a ‘one size fits all’ tool for general use [12]. Using a scoring system may result in earlier MTP triggering, but it is not clear if this may limit PRBC transfusion due to the attention to correcting coagulopathy, or whether in fact early MTP usage might risk overtransfusion due to the tendency for faster administration of PRBCs in the early stages of resuscitation. None of the studies included in the review by Shih et al. demonstrated that an MTP prediction tool improved patient outcomes. [12] Even in high volume trauma institutions transfusion management remains highly clinician dependent [13]. Experienced trauma surgeons struggle to accurately predict massive transfusion requirement early after arrival in the ED [14]. For now, it is likely that clinicians will continue to use a combination of clinical acumen, laboratory values, scoring systems, and imaging results. Overall, it is questionable if we need to predict the need for the use of MTP at all when resources are available and the blood and blood products can be expeditiously mobilised.

While transfusion triggers remain ill-defined and vary by institution and clinician, the decision to cease transfusion is also not rigidly established in the literature. Endpoints for PRBC transfusion are not mandated by our institutional MTP, though it does include targets of pH greater than 7.2, base deficit less than 6 and lactate less than 4 mmol/L.

Thus the decision to cease PRBC transfusion in early resuscitation is again made clinically and is informed by whether there has been achievement of haemostasis, anticipated ongoing blood loss, improving acidosis or base deficit, clinical markers of perfusion (BP, heart rate, urine output, mentation and skin examination) and Hb > 100 g/L.

The study is limited by the fact that it relies on retrospective analysis of registry data and the medical records. There are minimal clinically useful differences that reach significance, likely reflecting the small sample size, even in one the highest volume trauma centre in our state. The definition of overtransfusion is arbitrary and may not reflect clinically significant overtransfusion in all patients. This can make the overtransfusion problem even more negligible.

In conclusion, overtransfusion happened in over of a quarter of our transfused patients. There were no significant differences identified in these patients that were clinically relevant. The utilisation of MTP was not associated with higher overtransfusion rates. This study did not demonstrate any association between overtransfusion and mortality, length of stay or multi-organ failure.

Overtransfusion of red cells early in trauma is a commonly raised, but poorly documented concern. The number of patients in this study and the methodology prevent us making specific recommendations. Rather we would encourage other institutions to analyse their transfusion practice, so that collectively we can gather enough data to better understand the problem and define the equipoise between under- and over-transfusion in these patients.

## Data Availability

Available on request.
